# Ultrathin picoscale white light interferometer

**DOI:** 10.1038/s41598-022-12620-8

**Published:** 2022-05-23

**Authors:** Sunil Dahiya, Akansha Tyagi, Ankur Mandal, Thomas Pfeifer, Kamal P. Singh

**Affiliations:** 1 Department of Physical Sciences, Indian Institute of Science Education and Research Mohali, Sector 81, Mohali, 140306 India; 2 Max Planck Institute for Nuclear Physics, 69117 Heildelberg, Germany

**Keywords:** Optical techniques, Lasers, LEDs and light sources

## Abstract

White light interferometry is a well established technique with diverse precision applications, however, the conventional interferometers such as Michelson, Mach-Zehnder or Linnik are large in size, demand tedious alignment for obtaining white light fringes, require noise-isolation techniques to achieve sub-nanometric stability and importantly, exhibit unbalanced dispersion causing uncertainty in absolute zero delay reference. Here, we demonstrate an ultrathin white light interferometer enabling picometer resolution by exploiting the wavefront division of a broadband incoherent light beam after transmission through a pair of micrometer thin identical glass plates. Spatial overlap between the two diffracted split wavefronts readily produce high-contrast and stable white light fringes, with unambiguous reference to absolute zero path-delay position. The colored fringes evolve when one of the ultrathin plates is rotated to tune the interferometer with picometric resolution over tens of μm range. Our theoretical analysis validates formation of fringes and highlights self-calibration of the interferometer for picoscale measurements. We demonstrate measurement of coherence length of several broadband incoherent sources as small as a few micrometer with picoscale resolution. Furthermore, we propose a versatile double-pass configuration using the ultrathin interferometer enabling a sample cavity for additional applications in probing dynamical properties of matter.

## Introduction

White light interferometers are key tools for non-invasive and non-contact measurement of surface topography via vertical scanning interferometry, thin-film characterization, dispersion measurement of optical components and to characterize coherence properties of optical sources^1–7^. The white light interferometers are generally based on either amplitude division or wavefront division of a light beam^8^. To obtain white light interference with such interferometers, the optical path difference (OPD) between its two arms must be matched to well within the coherence length of the broadband source, which is typically a few optical cycles^9,10^. Amplitude division based interferometers such as Michelson, Mach-Zehnder, Mirau or Linnik setups^8,11^ are multi-component systems where it becomes challenging to achieve repeatable auto-reference to absolute zero delay position. In addition, the inteferometer must be stabilized against various acoustic, mechanical or other noises using active or passive approaches which make these systems large in size with tedious alignment. Although, picoscale resolution has been demonstrated previously with compact laser interferometers scanned via piezo-translation stages^12–14^, few works have achieved picoscale resolution and stability with a white light interferometer. Furthermore, to make quantitative measurements, it is essential to balance material dispersion in a white light interferometer along with an unambiguous reference to an absolute zero path difference which is difficult with conventional designs. The zero path delay position is an essential reference of an interferometer and is usually estimated using time domain analysis such as envelope’s amplitude method using white light interferograms^15^.

Previously, wavefront-division interferometers such as the classic Young’s double slit or Fresnel bi-prism have been used to obtain static white light fringes, however, without much tunability^8,16,17^. Although, tunable interferometers have been designed exploiting wavefront division by plane, spherical or toroidal split-mirrors, or ultra-thin glass plates, these have been mostly used with coherent ultrafast pulses as optical delay lines for pump-probe spectroscopy^18^. Split-mirror based designs do not straightforwardly produce white light interferograms with incoherent light due to intrinsic microscale coherence length of the broadband light sources, path-length fluctuations of the interferometer and a lack of picoscale tunability. One may wonder whether it is possible to design a compact and tunable wavefront splitting white light interferometer offering picoscale stability and resolution with unambiguous reference to absolute zero-path delay.

Here, we introduce a compact white-light interferometer exploiting wavefront division by a pair of identical ultra-thin glass plates with absolute zero delay reference and picometer resolution over tens of micrometer delay range. Our interferometer is auto-aligned to absolute zero path difference with perfect dispersion balance, thereby readily generating white light color fringes with broadband (quasi)-thermal sources. A theoretical analysis of the interferometer validates formation of high-contrast fringes along with its picoscale self-calibration for quantitative measurements. We measured the coherence length of many broadband sources having coherence length as small as a few micron with picoscale resolution. Furthermore, we show a double-pass setup using the ultrathin interferometer enabling a flexible sample-cavity for additional applications for probing dynamical properties of matter.

### Experimental setup

**Figure 1:**
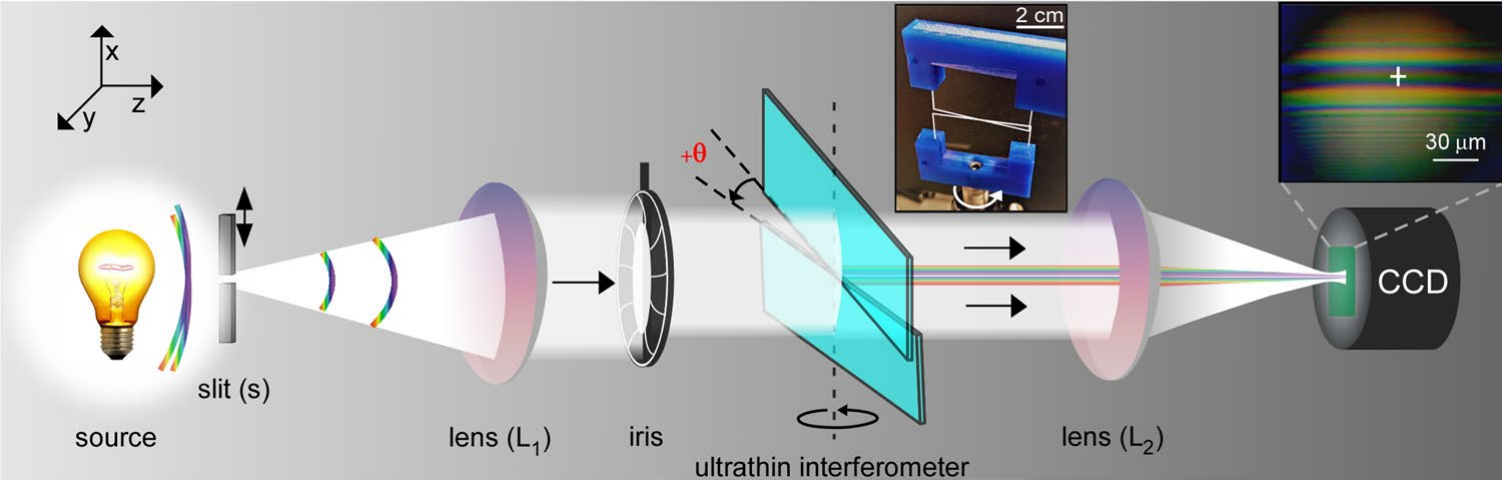
A schematic diagram of ultrathin white light interferometer. A broadband light source is made to pass through a variable slit (s) and collimated using a lens ($L_1$) and the interference fringes are collected on a chip with a convex lens ($L_2$). Insets: Pictures of the interferometer and a typical interference fringes.

**Figure 2:**
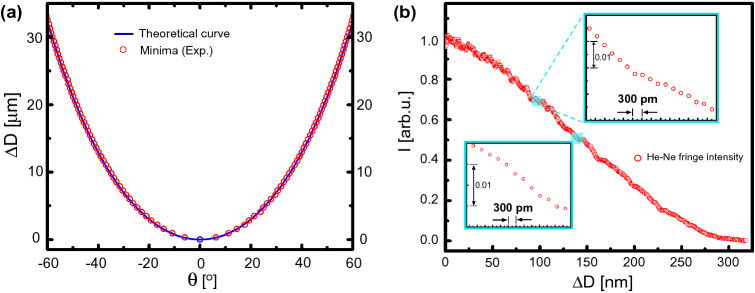
(**a**) Calibration of optical path delay $\Delta D$ vs rotation angle $\theta$ for t = 140 μm and n = 1.516. Solid line is theoretical curve using Eq. (4) and experimental data corresponds to minima values of He–Ne laser interferogram. (**b**) Experimental data showing self-calibrated picoscale resolution from bright to dark fringe tuning. The intensity variation near zero position is attributed to the various noises in the interferometer.

A schematic diagram of the ultrathin interferometer setup is shown in Fig. 1. The key component of the interferometer is a pair of identical rectangular-shaped, transparent and ultrathin glass plates, each of thickness t = 140 μm. Both the plates are vertically aligned in the xy-plane perpendicular to the direction of propagation of an incoming light (along the z-axis). The ultrathin glass plates were carefully yet firmly mounted in a 3d-printed frame and remained flat, as verified by optical profilometry^19^. The white light from an extended source is coupled into the interferometer via a rectangular micro-slit (slit width s = 200 μm, length = 1cm) and a collimating lens *L*_1_. The two ultrathin plates symmetrically split the incoming wavefront of the white light into an upper and a lower half, which overlap in the central region, thereby producing colored interference fringes. The interference fringes were captured through a lens (*L*_2_) directly on the CCD chip of a color camera and visualized/recorded on a computer. It is worth mentioning that the angular resolution of our lens-chip imaging was about 16 μrad (see Methods), which is much better than that of a naked human eye ($\sim 300$ μrad)^20^. This facilitates easy and high resolution recording of the white light fringes. We tuned relative optical path of the interferometer by finely rotating the lower ultrathin glass plate around a vertical axis passing through the centre of both the plates. The plate was rotated with a constant angular velocity of 0.5°/s with angular resolution corresponding to about 300 pm in the optical path-length delay near zero-delay position. We readily observed stable high-contrast colored fringes (typical visibility $\sim 0.8$) as shown in Fig. 1. The color of the central fringe changed deterministically as the interferometer was tuned which was recorded for subsequent analysis (see Supplementary video). The optical path resolution of our interferometer was around 300 pm, as depicted in linear region of fringe oscillation as shown in Fig. 2b. The range of optical path difference was from zero to 32 μm for the maximum rotation angle of *θ* = 60° which was chosen to be largely sufficient to estimate coherence length of most broadband white light sources.

Two key advantages of using a pair of micrometer thin glass plates in the interferometer are worth highlighting. First, due to the microscale thickness, these plates introduce negligible dispersion to the white light after transmission. Second, their fine rotation allows picoscale control and stability of the relative optical path-length yet offering tens of micrometer total delay range.

To prove the negligible dispersion effects in the ultrathin interferometer, we estimated the first and second-order dispersions in the ultrathin BK7 glass (t = 140 μm). The first-order dispersion was $dn/d\lambda \sim 0.04$ μm^-1^ for the glass^21^. The group delay dispersion (GDD)^21^ [GDD = t × GVD = $t\times (\lambda ^3/2\pi c^2)(d^2 n/d\lambda ^2)$] was about 9.8 $\hbox {fs}^2$ at 0° and 10.29 $\hbox {fs}^2$ at 30°, leading to a very small change in GDD value of below 0.5 $\hbox {fs}^2$ suggesting negligible dispersion effects. The dispersion introduced by the propagation of white light through air in our design can be safely ignored since the GVD value of air, approximately 0.02 $\hbox {fs}^2$/mm is much smaller^22^ and remains identical for both split beams. These values are much less compared to the conventional single or multi-pass white light interferometers such as the Michelson interferometer using mm-thick beam splitter. The axial chromatic aberration due to collimating/imaging lenses ($L_1$ and $L_2$) adds only a static background color distribution on the screen which hardly affect the resolution of the interferometer as determined by the minimum step size of the optical path length.

### Theoretical analysis

Using the Huygens-Fresnel-Kirchhoff formalism^23,24^, we have modelled the ultrathin interferometer and numerically obtained the interference fringes for monochromatic and broadband sources which were directly compared with the experiment. The ABCD matrix formalism was used to model diffraction and propagation of beam through free-space, focusing optics and the ultrathin glass plates^25^. The two vertically aligned glass plates act as two independent knife edges, that symmetrically split the incident wavefront to produce mirror-symmetric diffraction patterns which partially overlap in the central region. The Huygens-Fresnel-Kirchhoff equation is solved for both the co-propagating arms of the interferometer separately and the x-y limits were taken according to the size of the glass plates, including the vertical air-gap between them (which was experimentally adjusted close to plate thickness *t* ). The electric field amplitude in the observation plane is given by

1 $$E(x,y,z) = \left( {\frac{{ik}}{{2B\pi }}} \right)\exp ( - ikz)\int {\int {\exp } } \frac{{( - ik)\left[ {\left( {A\left( {x_{0}^{2} + y_{0}^{2} } \right)} \right) - 2x_{0} x - 2y_{0} y + D\left( {x^{2} + y^{2} } \right)} \right]}}{{2B}}U\left( {x_{0} ,y_{0} } \right)dx_{0} dy_{0} ,$$

where z is the propagation distance, k is the wave vector, $(x_0, y_0)$ and (*x*, *y*) are the coordinates at input and output planes, respectively. A, B and D are the matrix elements of the interferometer system in the beam path.

The input Gaussian beam $U(x_0,y_0)$ at $z=0$, within the paraxial approximation, is given as

2 $$U\left( {x_{0} ,y_{0} } \right) = \frac{{E_{0} w_{0} }}{{w(z)}}\exp \left( { - \frac{{ikA\left( {x_{0}^{2} + y_{0}^{2} } \right)}}{{2R(z)}} + i\phi (z)} \right)\exp \left( { - \frac{{ikA\left( {x_{0}^{2} + y_{0}^{2} } \right)}}{{w^{2} (z)}}} \right)\exp ( - ikz),$$

where $E_0$ denotes the electric field amplitude, *k* is the wavevector, $\phi (z)$ is the Gouy phase, $w_0$ is the beam waist, *w*(*z*) and *R*(*z*) are the beam radius and curvature, respectively. The spatial superposition of the two diffracted beams in the central region produces straight-line fringes, which evolve dynamically as the path-length is varied. The total electric field can be described as follows

3 $$\begin{aligned} E(x,y,z, \theta )=E_1(x,y,z)+E_2(x,y,z)exp{(ik\Delta D(\theta ))}, \end{aligned}$$

where $E_1(x,y,z)$ and $E_2(x,y,z)$ are the electric fields after propagation through upper and lower glass plates, respectively. $\Delta D(\theta )$ denotes the angle dependent path difference introduced by the lower glass plate. Upon rotation of the lower glass plate by an angle $\theta$, the light travels an extra optical path in the glass, which for a plate of refractive index *n* and thickness *t*, is given as^26–28^,

4 $$\Delta D(\theta ) = t\left[ {\sqrt {n^{2} - \sin ^{2} \theta } - \cos \theta - (n - 1)} \right].$$

The optical path difference is nonlinear w.r.t. $\theta$ as shown in Fig. 2a. For small angles, performing the Taylor’s expansion of the above Eq. (4) up to second order we obtain, $\Delta D (\theta ) \simeq [(n-1)/(2n)]t \times \theta ^2$. This parabolic non-linearity enables high resolution, which is around 300 pm in our case, as shown in Fig. 2b. Importantly, for parallel plates, the optical path length is zero and the dispersion is perfectly balanced in both the arms, which makes our interferometer unique with added advantages of simple alignment and operation.

The resultant intensity detected by a group of pixels centred at ($x_p,y_p$) covering an area $\delta A = \delta x \delta y$ will be $I = \int \left| E(x_p,y_p,z)\right| ^2 \delta A$. The corresponding experimental intensity is given as,

5 $$\begin{aligned} I(\theta ) = I_{0}\cos ^{2}[ k \Delta D(\theta )] \end{aligned}$$

where $k=2\pi /\lambda$ and $I_0$ is the maximum intensity detected by the single pixel.

**Figure 3:**
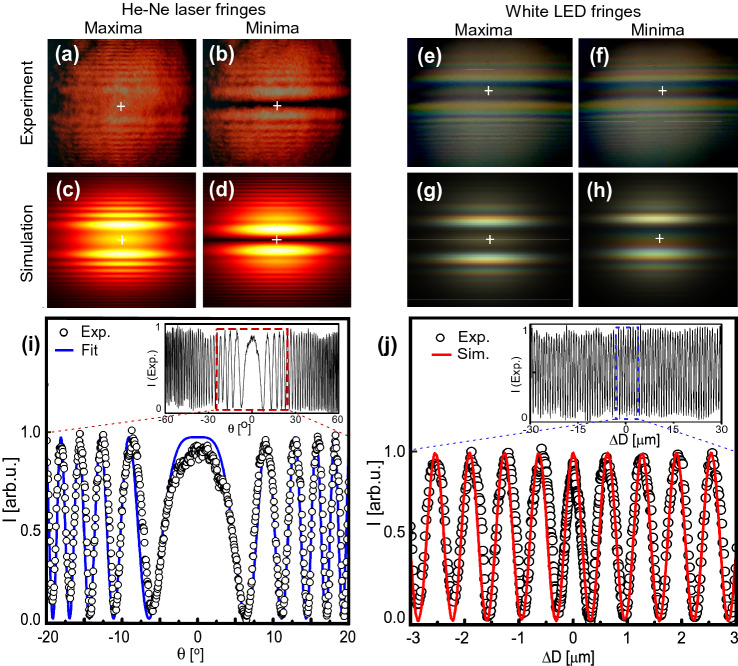
(**a**)–(**d**) Comparison of experimental and simulated interference fringes for a He–Ne laser, and (**e**)–(**h**) for white LED. The central maximum and minimum correspond to bright and dark central fringe. (**i**) Experimental intensity at cross-hair position along with a theoretical fit (Eq. 5) with $I_0$=1. Inset: experimental He–Ne laser intensity for larger angular range from $-60^\circ$ to $60^\circ$. (j) Simulated intensity and experimental intensity corresponding to (i) when calibrated in terms of optical path delay from -3 $\mu$m to 3 $\mu$m range. Inset: He–Ne laser intensity over a larger delay range from $-30~\mu m$ to $30~\mu m$.

For our experimental geometry (focal length, propagation distance, *t*), we first simulated interference fringes with a standard monochromatic He–Ne laser. Snapshots of fringes at two values of path lengths corresponding to central maximum and minimum are shown in Fig. 3c–d, in good agreement with the experiments (Fig. 3a–b). In addition, the simulated interferogram of He–Ne laser source is in good agreement with experimental interferogram as shown in Fig. 3j. The same formalism was also used to compute the interference pattern produced by a broadband white light source (see Methods for details) which is also in good agreement with the experiments as shown in Fig. 3e–h.

### Picoscale self-calibration and absolute zero optical path reference

A calibration of optical path delay in the interferometer is essential for quantitative applications. To validate our self-calibration of the path delay using the intensity of interference fringes, we first used a monochromatic He–Ne laser. Using the experimental parameters ($\lambda =632.8$ nm, n = 1.516 and t = 140 μm), $\theta$ was converted into optical path difference according to Eq. (4). As $\theta$ is varied, the central fringe intensity $I(\theta )$ oscillates (Fig. 3i). This allowed the interferometer to self-calibrate $\theta$ in terms of the displacement using the interference condition, i.e., the central maximum becomes minimum when the optical path difference of $\Delta D = \lambda /2$ is introduced by means of rotating the glass plate. The accuracy of the self-calibration process is determined by the precision of the wavelength of the monochromatic laser source which in our case was about 100 pm. The resolution of our interferometer is determined by the OPD corresponding to the minimum repeatable step size of the rotation stage. A resolution of about 300 pm was obtained with a simple camera/photodiode without any signal processing or complex noise-isolation approaches (Fig. 2b). The 300 pm optical path change corresponds to $\Delta \theta = 0.22$° near zero path delay and $\Delta \theta = 0.012$° near $4$° rotation angle (see Methods). This performed calibration of the interferometer remains valid for the broadband white light source^6^. It is worth highlighting that due to the micrometer thickness of the plates, our interferometer possesses a unique advantage of achieving absolute zero path difference with perfect dispersion balance when both the plates are parallel. In fact, the error in parallelism between the two plates was below 0.05°, which is negligible since the corresponding uncertainty in the optical path delay is much smaller than one optical cycle ($\lambda /1000$ and corresponding temporal uncertainty of a few as). Therefore, the absolute zero delay position can be easily found directly by just keeping both the plates parallel. Moreover, by rotating the plate in both the positive and negative directions, one can further refine the zero position by exploiting the symmetric dependence of $\Delta D(\theta )$ around $\theta =0$ (see Fig. 2a). In our case, a large variation in $\theta \sim 7^\circ$ is needed in order for the central maximum to become the first minimum with He–Ne laser in Fig. 3i. Hence, attaining zero path-delay position is straightforward and cannot be missed in practical situations for any broadband incoherent source.

### Measurement of micro-scale coherence length of broadband light sources

**Figure 4:**
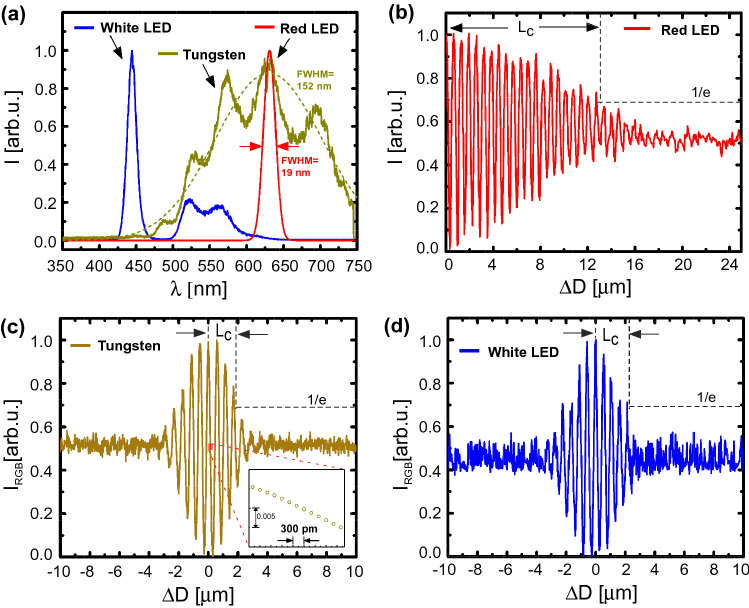
(**a**) Measured spectra of three different light sources. Experimental interferogram of (**b**) red LED, (**c**) tungsten bulb and (**d**) white LED. The corresponding coherence length $L_c$ is labelled. Inset in (**c**) shows picoscale resolution.

Broadband sources such as tungsten bulbs and white LEDs have very low temporal coherence of a few optical cycles long and its precision measurement demands nanometer control and stability in optical path. Using three different broadband sources, we easily obtained their corresponding interferograms as shown in Fig. 4. These sources were collimated by a slit (s = 200–500 μm) and a lens ($L_1$) while a second iris controlled the beam size before the ultrathin interferometer. The slit improved the spatial coherence of the source without altering its spectrum or temporal coherence length (Supplementary Fig. S1)^29,30^. The fringes and interferogram for each source at absolute zero delay [$\Delta D(\theta =0)=0$], where all the spectral components interfere constructively, are shown in Supplementary Figs. S2 and S3. The central fringe intensity vs optical path delay ($\Delta D$) for red LED, tungsten lamp and white LED are shown in Fig. 4b–d, respectively.

From the interferogram, the coherence length ($L_c$) is defined as the OPD at which the fringe envelope becomes 1/e of the maximum value^31,32^. One can also calculate the corresponding coherence time of the source as $t_c$ = $L_c/c$, where *c* is the speed of light in vacuum. For the red LED (central $\lambda _0 = 631$ nm, FWHM bandwidth $\Delta \lambda =19$ nm), the coherence length was $L_c= 13.86$ μm. This value was theoretically calculated assuming a Gaussian spectrum of light by^33^ $\left( {L_{c} = \sqrt {\frac{{2\ln 2}}{\pi }} \frac{{\lambda _{0}^{2} }}{{\Delta \lambda }}} \right)$. The experimental value from the interferogram of $L_c= 13.02 \pm 0.04$ μm was in reasonable agreement with the theoretical estimate within $6\%$ error. For the tungsten bulb, the $L_{c}$ was measured to be 1.92 ± 0.029 μm which was also in reasonable agreement with the theoretical estimate of 1.75 μm calculated assuming a Gaussian spectrum of light of identical bandwidth as shown in Fig. 4a. Similarly, coherence length of white LED source is found to be 2.22 ± 0.035 μm. Although, the actual emission spectra of these sources were not Gaussian, the above reasonable agreement supports our measurements. It is worth highlighting that the picoscale resolution and stability of the ultrathin interferometer facilitates reliable detection of broadband interferograms down to a few-cycles width with absolute zero path difference reference.

## Discussion

**Figure 5:**
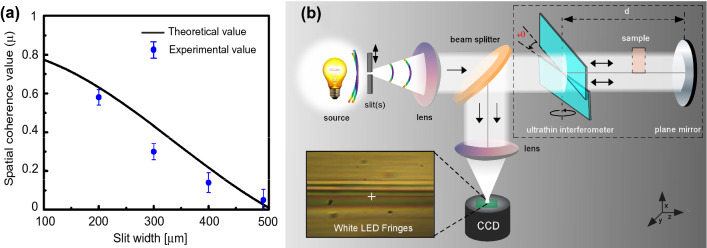
(**a**) Effect of slit width (s) on spatial coherence value ($\mu$) or visibility (V) of fringes of white LED source. Theoretical curve corresponds to A$\left| \sin (\frac{\pi us}{\lambda L})/(\frac{\pi us}{\lambda L})\right|$ with experimental parameters u = 250 μm, $\lambda$= 440 nm, L = 32 cm and A = 0.83. (**b**) Schematic of double pass ultrathin interferometer. Inset: white LED interference fringes are shown.

We propose a versatile double-pass design exploiting our ultrathin interferometer that allows a ’sample-cavity’ for placing a sample (film, gas-medium, flame) under investigation as shown in Fig. 5b. While the basic working principle of the interferometer is the same as described before, here the interference condition is modified to account for two passes of light through the ultrathin glass as, $I(\theta ) = I_{0}\cos ^{2}[ 2 k \Delta D(\theta )]$. Inset in Fig. 5b shows stable fringes obtained with the setup. Furthermore, for a given geometry, the contrast of fringes can be improved by optimizing the slit-width (*s*) which controls the spatial coherence of the light as, $\mu = \left| \sin (\frac{\pi us}{\lambda L})/(\frac{\pi us}{\lambda L})\right|$, where L is distance between the slit and interferometer and *u* is the central gap between the two glass plates. Figure 5a shows our measurement of spatial coherence ($\mu$) versus slit width keeping other parameters fixed. Narrowing the slit width improves the contrast^29^, but reduces the light throughput. In practical situations, *s* should be appropriately adjusted to achieve a sufficiently high fringe intensity as well as high fringe contrast. Furthermore, to achieve the high fringe contrast, the gap between the glass plates (*u*) should be minimum to ensure the maximum spatial overlap between the two split wavefronts of the transmitted light because the light passing through the central gap adds a non-interfering background contribution. The stabilty of our interferometer in the sub-nanometer scale is limited by various noises caused by the repeatable step-size of the servomotor, intensity fluctuations of the laser and noise in the detector (photodiode/CCD). Employing intensity stable lasers, cooled detectors and better rotation stage could further improve the noise-limited resolution.

One more aspect of this interferometer is worth discussing which relates to a transverse displacement of a light beam after passing through a tilted plate given as^34^, $\delta (\theta ) = [(n-1)t/n]\times \theta$ . For single pass, the maximum lateral shift is $\delta = 72$ μm for $\theta =60^\circ$ which is negligible compared to the beam size $\sim 5$ mm. Furthermore, since we directly capture the fringes on a chip located near the focus of the lens ($L_2$), the effect of $\delta$ translates into an angular deflection which is negligible in the focal plane. In the case of the double-pass design of Fig. 5b, the net transverse displacement after back and forth passes is completely eliminated.

## Conclusions

We have established a new wavefront splitting ultrathin glass based picoscale white light interferometer. Our interferometer is easy to align, provides a direct and unambiguous reference to absolute zero path delay with dispersion balanced arms with picoscale resolution and stability. A good agreement between the experimental interferograms and theoretical simulations validates our design and its self-calibrated approach for picoscale measurements. We readily produced white light fringes from three different broadband sources (a tungsten lamp, white LED and red LED) and measured their coherence length as small as a few micrometers. Furthermore, we propose a double-pass configuration of our interferometer which allows placing of various samples for precise measurement of dynamical as well as static properties of matter. It should be possible to further reduce static chromatic aberration introduced by replacing the refractive optics with collimating/focusing mirrors.

Multiple applications are envisioned, for example, in measurement of optical properties of biological thin films and solutions^35^, linear or non-linear refractive index of semi-transparent solids and optical density fluctuation of air due to humidity, turbulence and temperature^8^. Recent availability of highly transparent, optical quality (1 nm rms roughness) glass with inch-scale size and thickness down to 30 μm could further enhance the resolution and dispersion management of such ultrathin interferometers^36^. It should also be possible to couple the interferometer with an optical fiber probe for applications demanding remote operation.

## Methods

### Fabrication of ultrathin interferometer

Two identical rectangular ultrathin glass plates of borosilicate material (BK7) having thickness t = 140 μm and refractive index n = 1.516 (for 632 nm wavelength) was used. The size of the plates was 22 mm $\times$ 40 mm. In order to mount these plates without any bending and stretching, a U-shaped plastic mount with dedicated groves for holding the ultrathin plates were 3-D printed. The lower plate was mounted on a motorized rotation stage (Thorlabs PRM1Z7) while the upper plate was fixed. The resolution of the rotation stage is 0.0003° (1 arcsec) while moving in one direction. The lack of curvature in the mounted ultrathin plates was verified by far-field beam profilometry using a He–Ne laser^19^. The vertical gap between the plates was uniformly minimized to about thickness of glass plate by using the same ultrathin glass plate as a removable spacer between the upper and lower plates.

### Simulation of white-light fringes

To numerically compute the interference fringes for a broadband light source, we first measure the emission spectrum of source $S(\lambda )$. The measured spectrum is decomposed in three spectral regions corresponding to red, green and blue to obtain the relative intensity distributions denoted as, $S_R({\lambda }),~S_G({\lambda }), ~S_B({\lambda }$) over an entire wavelength range (350–750 nm). Each colored region was further sampled at a wavelength interval of 10 nm, in effect decomposing the broadband spectrum into N monochromatic components ($\lambda _N$) of different strengths. For each component $\lambda _N$, the Huygens-Fresnel-Kirchhoff equation was solved to compute the electric field $E_{\lambda _N} (x,y)$ which was used to obtain the corresponding intensity $(I_{\lambda _N} (x,y)= \left| E_{\lambda _N}(x,y)\right| ^2)$ according to Eq. (3). These intensities were weighted as per the experimental spectrum, $S_R({\lambda }),~S_G({\lambda }), ~S_B({\lambda }$) via relation $I_{R} = S_R({\lambda })\times I_{\lambda }(x,y)$ and similarly, $I_{G} = S_G({\lambda })\times I_{\lambda }(x,y)$, $I_{B} = S_B({\lambda })\times I_{\lambda }(x,y)$. Finally, by adding together the individual numerical diffraction patterns, we obtained the interference pattern for the broadband source in the xy-plane at specific z value.

### Data acquisition and analysis

The rotation of the lower glass plate was controlled using Thorlab’s APT software (version 3.21.5, https://www.thorlabs.com/newgrouppage9.cfm?objectgroup_id=9019) by rotating a servo controlled stepper motor. The total angular range, angular velocity and dwell time can be modified to capture the dynamics of the interferogram appropriately. The typical angular speed was 0.5$^\circ$/sec and the dynamic fringes were video recorded at 25 fps with a camera (Thorlabs DCC1645C). The chip size was 4.6 mm $\times$ 3.7 mm and the single-pixel size of about 4 $\times$ 4 μm^2^. The angular resolution of the chip-lens system (pixel size/f) for f = 25 cm was about 16 μrad. The videos containing interference fringes were analysed for intensity variation in the area of around 3 pixels radius using Tracker software (version 5.1.5, https://physlets.org/tracker/). The background noise intensity was subtracted from the interferogram of tungsten and white LED and the maximum intensity was normalized to unity.

## Supplementary Information

Supplementary Information 1.

Supplementary Information 2.

Supplementary Information 3.
