# Supplementary material for: Ultrathin picoscale white light interferometer
Source: Sci Rep. 2022 May 23;12:8656. doi: 10.1038/s41598-022-12620-8 (PMC9126962; doi:10.1038/s41598-022-12620-8)
Supplement: Supplementary file 1 — Supplementary Information 1. [file 41598_2022_12620_MOESM1_ESM.pdf]

Supplementary information for

Ultrathin picoscale white light interferometer

Sunil Dahiya[1], Akansha Tyagi[1], Ankur Mandal[1], Thomas Pfeifer[2], and Kamal P. Singh[1*]

[1]*Department of Physical Sciences, Indian Institute of Science Education and Research Mohali, Sector 81, Mohali 140306, India*
[2]*Max Planck Institute for Nuclear Physics, 69117 Heildelberg, Germany*
**corresponding author: kpsingh@iisermohali.ac.in*

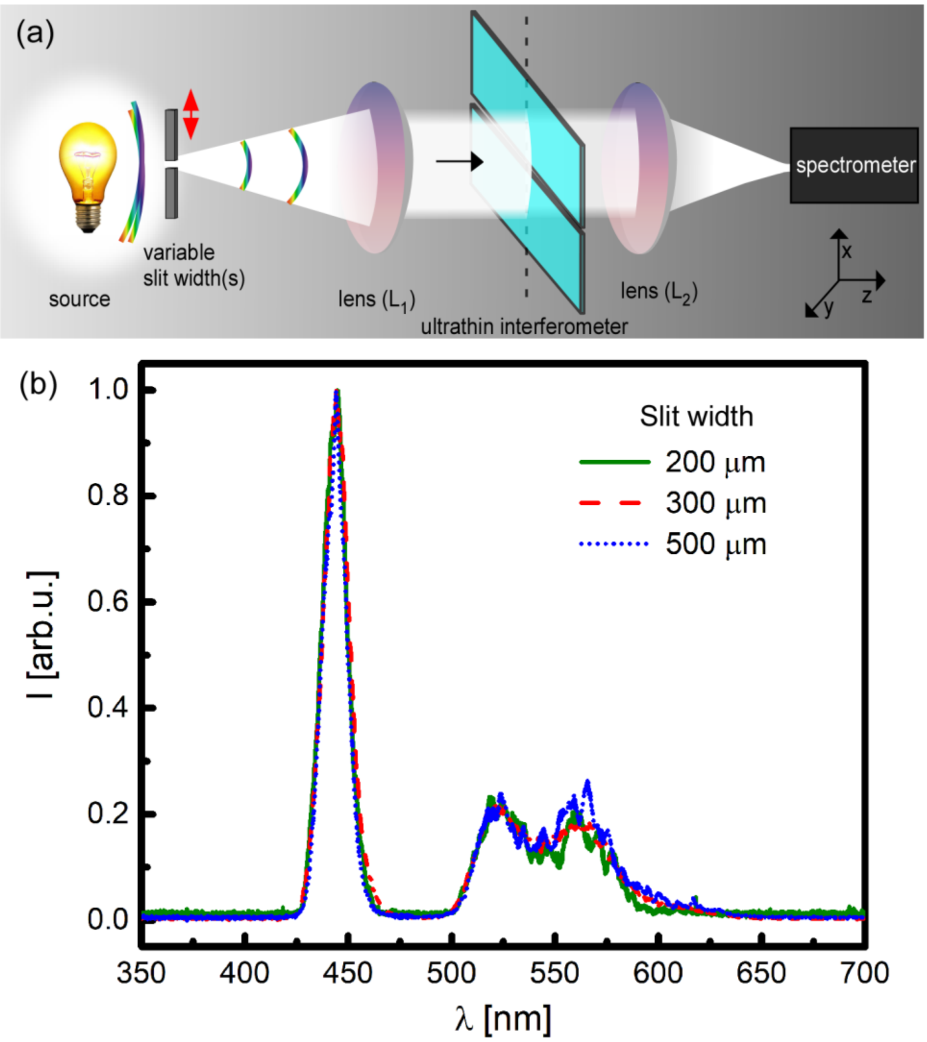

Fig. S1. (a) Setup for emission spectrum measurement of white LED source with variation of slit width. (b) Spectrum of white LED with variation of slit width is shown here. Spectrum is normalized to unity (for maximum intensity peak around 450 nm) for each slit width. From graph, emission spectrum of source does not change with slit width variation and hence the temporal coherence length remains same.

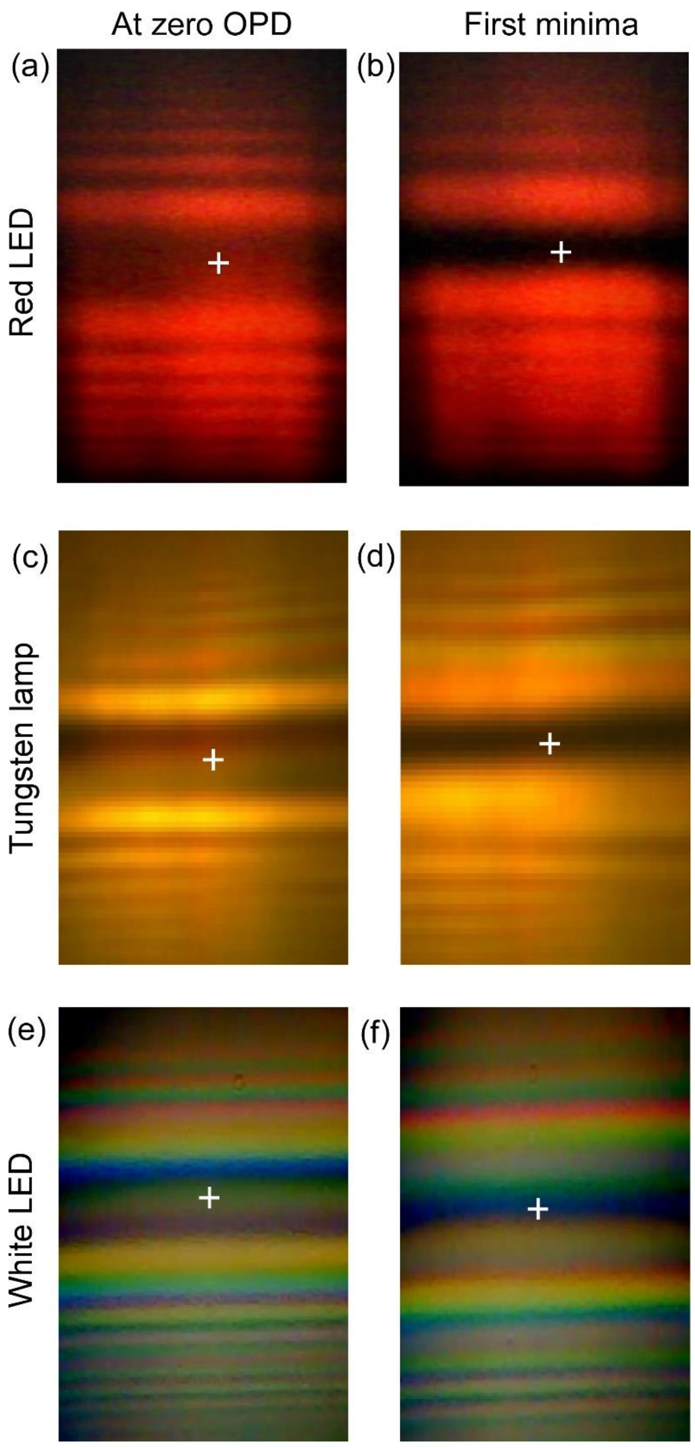

Fig. S2. Images of red LED, tungsten and white LED source fringes captured at CCD chip using ultrathin glass interferometer, showing constructive interference (maxima) at zero OPD and destructive interference (first minima). The cross mark shows the intensity tracking position for interferogram.

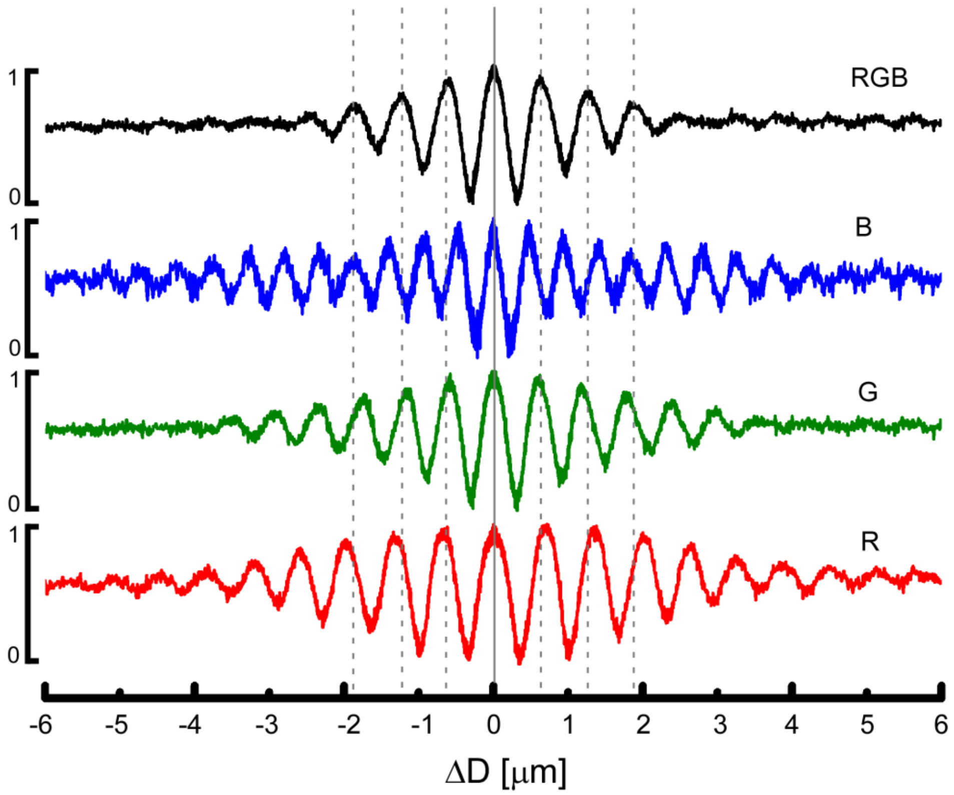

Fig. S3. Experimental white light interferogram (RGB) along with its R, G and B components. All spectral components (R, G, B) interfere constructively (in phase) at zero path difference position.
