# Supplementary material for: Ultrathin picoscale white light interferometer
Source: Sci Rep. 2022 May 23;12:8656. doi: 10.1038/s41598-022-12620-8 (PMC9126962; doi:10.1038/s41598-022-12620-8)
Supplement: Supplementary file 2 — Supplementary Information 2. [file 41598_2022_12620_MOESM2_ESM.pdf]

# Supplementary video information

# Ultrathin picoscale white light interferometer

Sunil Dahiya[1], Akansha Tyagi[1], Ankur Mandal[1], Thomas Pfeifer[2], and Kamal P. Singh[1*]

[1]*Department of Physical Sciences, Indian Institute of Science Education and Research Mohali, Sector 81, Mohali 140306, India*
[2]*Max Planck Institute for Nuclear Physics, 69117 Heildelberg, Germany*
**corresponding author: kpsingh@iisermohali.ac.in*

**Supplementary video S1:** Evolution of white LED fringes.
The video demonstrates evolution of central interference fringes of white LED made using ultrathin white light interferometer with glass rotation angle θ = $-26^0$ to $24^0$. The fringes were captured directly at camera chip.
